# Dental Treatment in a State-Funded Primary Dental Care Facility: Contextual and Individual Predictors of Treatment Need?

**DOI:** 10.1371/journal.pone.0169004

**Published:** 2017-01-24

**Authors:** Kristina L. Wanyonyi, David R. Radford, Jennifer E. Gallagher

**Affiliations:** 1 University of Portsmouth Dental Academy, Hampshire Terrace, Portsmouth, United Kingdom; 2 King's College London Dental Institute, Population and Patient Health Division, London, United Kingdom; 3 King's College London Dental Institute, Teaching Division, Guys Tower, Guys Hospital, London, United Kingdom; University of Washington, UNITED STATES

## Abstract

**Objective:**

This study examined individual and contextual factors which predict the dental care received by patients in a state-funded primary dental care training facility in England.

**Methods:**

Routine clinical and demographic data were extracted from a live dental patient management system in a state-funded facility using novel methods. The data, spanning a four-year period [2008–2012] were cleaned, validated, linked by means of postcode to deprivation status, and analysed to identify factors which predict dental treatment need. The predictive relationship between patients’ individual characteristics (demography, smoking, payment status) and contextual experience (deprivation based on area of residence), with common dental treatments received was examined using unadjusted analysis and adjusted logistic regression. Additionally, multilevel modelling was used to establish the isolated influence of area of residence on treatments.

**Results:**

Data on 6,351 dental patients extracted comprised of 147,417 treatment procedures delivered across 10,371 courses of care. Individual level factors associated with the treatments were age, sex, payment exemption and smoking status and deprivation associated with area of residence was a contextual predictor of treatment. More than 50% of children (<18 years) and older adults (≥65 years) received preventive care in the form of ‘instruction and advice’, compared with 46% of working age adults (18–64 years); p = 0.001. The odds of receiving treatment increased with each increasing year of age amongst adults (p = 0.001): ‘partial dentures’ (7%); ‘scale and polish’ (3.7%); ‘tooth extraction’ (3%; p = 0.001), and ‘instruction and advice’ (3%; p = 0.001). Smokers had a higher likelihood of receiving all treatments; and were notably over four times more likely to receive ‘instruction and advice’ than non-smokers (OR 4.124; 95% CI: 3.088–5.508; p = 0.01). A further new finding from the multilevel models was a significant difference in treatment related to area of residence; adults from the most deprived quintile were *more likely* to receive ‘tooth extraction’ when compared with least deprived, and *less likely* to receive preventive ‘instruction and advice’ (p = 0.01).

**Conclusion:**

This is the first study to model patient management data from a state-funded dental service and show that individual and contextual factors predict common treatments received. Implications of this research include the importance of making provision for our aging population and ensuring that preventative care is available to all. Further research is required to explain the interaction of organisational and system policies, practitioner and patient perspectives on care and, thus, inform effective commissioning and provision of dental services.

## Introduction

The World Health Organization (WHO) is promoting Universal Health Coverage (UHC) in support of achieving the 2030 Sustainable Development Goals [1]. Research within state-funded health systems such as the National Health Service (NHS) [2] in England provides evidence of how opportunities to access dental services free at the point of delivery (children and vulnerable adults) or at reduced cost (adults make a co-payment), align to oral health needs. There is clear evidence that variations in dental service uptake are associated with social status [3,4], with socially deprived groups accessing care less frequently [4], and requiring more extensive services such as treatment under sedation [5], when they do access care. These patterns of access further contribute to increased oral health need and inequalities across the socio-economic spectrum, despite the state-funded service provision [6]. What is unknown is how routine care provision relates to need once access to dental services is gained, and how this impacts on equity of health outcomes. This is particularly important for state-funded dental services such as the NHS which is committed to maximising preventative care [7], as well as equity and quality [8]. The NHS serves a large proportion of the national population with seven out of 10 children, and five out of 10 adults, attending primary dental care within a 24-month period [9]. So far the analysis of NHS data has predominantly been studied to monitor new initiatives [10], assess value for money [11], and the longevity of treatments [12–19], with much of this research conducted under previous models of care. A more analytical evaluation of dental activity from contemporary NHS primary care has the potential to provide information on how encounters with health care under the current system contribute to addressing oral health needs.

More specifically, dental activity records, which are routinely collected within patient management systems, could answer questions regarding what happens when patients enter primary dental care. Is patients’ treatment related to known patterns of need and health behaviours such as smoking, and how does this relate to NHS provisions to improve access such as payment exemption? Finally, this could further inform understanding of how care relates to contextual level predictors of oral health need. Thus patient management systems are potentially a rich data source consisting of patient demography, and care received experience. Information on patient’s residence is also available, and when augmented with census data, can provide information about the patients’ deprivation at area of residence [20], which represents a contextual variable. Obtaining data from patient management systems eliminates recall, selection and social desirability biases common to surveys; an alternative research method [21]. In the USA, dental claims data derived from patient management systems have been used to investigate patterns in children’s oral health [22]. In Ireland, similar data have been used to describe national trends in treatment requirements [23]. Whilst in the NHS in England, primary care data generated from these systems have been mainly used to monitor and negotiate contracts with providers, and only recently to predict future demand [11]. The literature to date is largely descriptive in nature; therefore, leaving the analytical potential of dental data underexplored. Thus, there is a need for research to mine routinely collected primary care data in order to understand patterns of dental care and advance in this field of research.

The objective of this study was to investigate the relationship between dental care received and both individual (age, sex, adult payment status and smoking behaviour) and contextual factors (deprivation in the patients’ immediate living environment) using routinely collected patient management data.

## Materials and Methods

Ethical approval for this research was provided by NRES Committee Fulham REC: Reference No. 11/LO/1138 Protocol No. NTMHWMOV3 and research governance approval by NHS Portsmouth R&D Committee Reference No. SSPS/05/11.

This research was conducted on patient care at the University of Portsmouth Dental Academy (UPDA), a state-funded NHS primary care service and training centre, where dental students on community outreach, and UPDA dental hygiene-therapy students, learn together in practice teams. There is a strong ethos on using the skill mix of the dental team and delivering contemporary evidence-based primary care. For two out of the four years under study, patients were treated at no cost. UPDA holds a dental contract with the NHS similar to practices across England, which requires performance targets to be achieved annually. To manage patient care the centre uses a live patient management system (Clinical +) developed by Carestream Limited [24,25], from which data for this study were extracted.

The extracted data constituted courses of care, which had either been completed or closed within the four-year period, including both emergency and planned care. Courses of care were ‘closed’ when patients failed to return for care but could not be distinguished from ‘completed’ courses in the system. The first two years involved dental hygiene-therapy students providing care supported by general dental practitioners, whilst the latter two years involved team training between dental and dental hygiene-therapy students, with care provided free at the point of delivery.

The data were cleaned and validated using a combination of techniques adapted from health services research and information technology [26–30]. The process identified data inputting errors and software design problems. After data were cleaned a validation analysis was undertaken to identify outliers and inconsistency in the data when compared to other locally generated reports. Within the extracted data multiple courses of care were included and each treatment received was linked to date of completion or closing of the care plan. This was further linked to the patient’s age, sex, payment-exemption status, postcode and smoking status. Smoking status was only available for patients who had been seen at least once in the two latter years. Postcode data were transformed using the Indices of Multiple Deprivation (IMD) to provide a contextual measure; area level deprivation. The latter is a measure used in England to provide a relative measure of deprivation at Lower Level Super Output Area (LSOA), which is an area of 1000–1500 households describing the cumulative deprivation score of individuals in the area [31]. Payment-exemption status was used in the analysis; it has been shown to correlate with income deprivation at the area level, but not overall deprivation at the area level [20]. Therefore, this was used as a measure of individual income deprivation in the analysis, together with the variable for overall contextual area level deprivation—identified as a patient’s quintile of deprivation–and derived from IMD [31].

Analysis was undertaken in five stages. First, descriptive analysis of patient demography was undertaken to provide insight to the patient base. Second, analysis of all treatments received and their rate of occurrence. Third, the five treatments with the highest volume of activity were further analysed against the socio-demography of patients using unadjusted analysis and chi-square tests of significance; to examine differences in treatment rates by socio-demography. The treatments included were i] ‘tooth restoration’, ii] ‘instruction and advice’ (information on brushing teeth and diet), iii] ‘scale and polish’, iv] ‘tooth extraction’, and v] ‘partial dentures’. For this unadjusted analysis, the age variable was converted to a categorical variable of three groups (< 18 years, i.e. children), 18–64 years (working-age adults) and 65 years and over (older people). This was in order to validate the data through outlining expected and established differences in cohort effect between these three sections of the population.

Fourth, adjusted logistic regression was used to examine predictors of common treatments while controlling for confounding variables. Five separate logistic regression models were undertaken. This involved five sub-sets of data, one for each treatment. Each dataset consisted of all adult patients, and the creation of a binary variable indicating whether they had or had not received specific treatments. Only adult patients were included in the logistic regression analysis to mitigate bias associated with non-fee-paying children. Age was then transformed to a continuous variable for this adjusted logistic regression analysis, as this second analysis was exploratory of *adults only*, and sought to investigate less established links between patient-age of adults and the range of treatment types. Receiver Operator Curve (ROC) was used to validate the models’ predictive power [0.5–0.6 weak; 0.6–0.7 fair; 0.8-and above strong model predicting power].

Fifth, and finally, multilevel modelling was undertaken. Logistic regression models which had indicated existing associations with area level measures of deprivation of p<0.05 (quintile of deprivation) were subjected to this analysis in order to establish whether there was grouping at the level of residence that predicted care. LSOAs were selected as the grouping variable, as this was the smallest aggregating variable available to be augmented to the data set [32]. This multilevel analysis was able to extend the regression analysis to a situation where data were hierarchical [33], and test the potential influence of unknown factors at the level of area of residence on health.

## Results

### Participants

The patient management dataset comprised 6,351 patients that had received 147,417 treatment procedures of care across 10,371 courses of care over the four-year study period [2008–2012]. All courses of care extracted were either completed or closed; the latter because the patient had not returned to complete care. The majority were adult (82%), male (52.2%), and non-smokers as shown in Table 1. The age range across the four-year period was 1–94 years and patients from the most deprived quintile (23.3%) exceeded those from the least deprived (11.9%).

**Table 1:** Individual and social characteristic of patients with closed/completed treatment plans between 2008/09 to 2011/12 academic years at UPDA.

Patient related variables		Frequency	%
**Adult exemption status (n = 5185)**	Exempt adult	1,005	19.4
	Non-exempt adult	4,180	80.6
**Age groups (n = 6,351)**	0–2yrs	85	1.3
	3–5yrs	247	3.9
	6–12yrs	541	8.5
	13–17yrs	274	4.3
	18–24yrs	1,211	19.1
	25–34yrs	1,272	20
	35–44yrss	1,008	15.9
	45–54yrs	813	12.8
	55–64yrs	494	7.8
	65–74yrs	260	4.1
	Over 75yrs	146	2.3
**Quintile of deprivation (n = 6259)**	Most deprived	1,477	23.3
	2	1,318	20.8
	3	1,414	22.3
	4	1,314	20.7
	Least deprived	736	11.6
**Sex (n = 6,351)**	Female	3,098	48.8
	Male	3,253	52.2
**Smoking status (n = 3436)**	Non-smoker	2,803	81.6
	Smoker	633	18.4

Note

n = 6,351 unless otherwise stated

Age groupings are based on state-funded banding

UPDA- University of Portsmouth Dental Academy

### Treatments and socio-demography

Of the 147,417 treatments delivered, the five most frequently occurring were: ‘tooth restoration’ (51.5%); ‘instruction and advice’ (49.2%); ‘scale and polish’ (38.7%); ‘tooth extraction’ (25.1%); and ‘partial dentures’ (5.1%).

### Unadjusted analysis (adults and children)

The results of the unadjusted analysis, which included adults and children (Table 2), suggest that the proportion of patients receiving at least one ‘tooth restoration’ in the four-year period significantly differed by age, sex and smoking status, but not by social factors (deprivation or payment status). Older (≥65 years) and working age adults (18–64 years) had a higher rate of ‘tooth restoration’ (54.4% and 54.3% respectively), exceeding the volume amongst children and young people (<18 years) (38%); p = 0.001. A higher proportion of males had received a ‘tooth restoration’ compared with females (54% cf 49%; p = 0.001). Interestingly, smokers had a higher proportion of patients with ‘tooth restoration’ compared with non-smokers (67% cf 54%; p = 0.01). In contrast to the above, ‘instruction and advice’ had been received by a higher proportion of adults who were non-exempt from payment compared with exempt (50.3% cf. 32.1%; p = 0.01. Similarly more adult patients from the least deprived areas compared with the most deprived had received ‘instruction and advice’ (53.9 cf 46.1%; p = 0.003), whilst more smokers had received ‘instruction and advice’ than non-smokers (90.2% cf 74.5%: p = 0.001). There was no significant difference in receipt of ‘instruction and advice’ by sex.

**Table 2:** Unadjusted model of proportion of patients who experienced a treatment by sociodemography between 2008/09 to 2011/12 academic years at UPDA.

	Patient related variables		Never received treatment within four academic years N (%)	Received treatment within four academic years N(%)	P value
**Tooth restoration**	**Overall**		3,083 (48.5)	3,268(51.5)	
	**Adult payment status(n = 5,185)**	**Exempt**	475 (47.3)	530 (52.7)	0.236
		Non-exempt	1,889 (45.2)	2291(54.8)	
	**Age groups (n = 6,351)**	Under 18	707 (61.6)	440 (38.4)	0.001*
		18–64 years	2,209 (45.7)	2,629 (54.3)	
		Over 65	167 (45.6)	199 (54.4)	
	**Sex (n = 6,351)**	Female	1,576 (50.9)	1,522 (49.1)	
		Male	1,507 (46.3)	1,746 (53.7)	0.001*
	**Quintiles of deprivation in PCT (n = 6,259)**	Most deprived1	741 (50.2)	736 (49.8)	0.139
		2	657 (49.8)	661 (50.2)	
		3	684 (48.4)	730 (51.6)	
		4	600 (45.7)	714 (54.3)	
		Least deprived 5	353 (48.0)	383 (52.0)	
	**Smoking cessation signposting (n = 3436)**	No	1304 (46.5)	1499 (53.5)	0.001*
		Yes	211(33.3)	422 (66.7)	
**Instruction and advice**	**Overall**		3,224 (50.8)	3,127(49.2)	
	**Adult payment status (n = 5,185)**	Exempt	682 (67.9)	283 (32.1)	0.01*
		Non-exempt	2,078 (49.7)	2,102 (50.3)	
	**Age groups (n = 6,351)**	Under 18	468 (40.8)	679 (59.2)	0.001*
		18–64 years	2,597 (54.1)	2,201 (45.9)	
		Over 65	159 (39.2)	247 (60.8)	
	**Sex (n = 6,351)**	Female	1,584 (51.1)	1,514 (48.9)	0.293
		Male	1,640 (50.4)	1,613 (49.6)	
	**Quintiles of deprivation in PCT (n = 6,259)**	Most deprived 1	796 (53.9)	681 (46.1)	0.003*
		2	676 (51.3)	642 (48.7)	
		3	721 (51.0)	693 (49.0)	
		4	631 (48.0)	683 (52.0)	
		Least deprived 5	339 (46.1)	397 (53.9)	
	**Smoking status (n = 3436)**	No	716 (25.5	2,087 (74.5)	0.001*
		Yes	62 (9.8)	571 (90.2)	
**Scale and polish**	**Overall**		3,890 (61.3)	2,461(38.7)	
	**Adult payment status (n = 5,185)**	Exempt	662 (65.9)	343 (34.1)	0.001*
		Non-exempt	2,199 (52.6)	1,981 (47.4)	
	**Age groups (n = 6,351)**	Under 18	1,028 (89.6)	119 (10.4)	0.001*
		18–64 years	2,717 (56.2)	2,121 (43.8)	
		Over 65	145 (39.6)	221 (60.4)	
	**Sex (n = 6,351)**	Female	2,390 (77.1)	708 (22.9)	0.398
		Male	1,998 (61.4)	1,255 (38.6)	
	**Quintiles of deprivation in PCT (n = 6,259)**	Most deprived 1	1,027 (69.5)	450 (30.5)	0.001*
		2	775 (58.8)	543 (41.2)	
		3	841 (59.5)	573 (40.5)	
		4	792 (60.3)	522 (39.7)	
		Least deprived 5	388 (52.7)	348 (47.3)	
	**Smoking status (n = 3436)**	No	1,466(52.3)	1337 (47.7)	0.001*
		Yes	201 (31.8)	432 (68.2)	
**Tooth extraction**	**Overall**		4754 (74.9)	1597 (25.1)	
	**Adult Payment status (n = 5,185)**	Exempt	622 (61.9))	383 (38.1)	0.001*
		Non-exempt	3,115 (74.5)	1,065(25.5)	
	**Age groups (n = 6,351)**	Under 18	1,002 (87.4))	145 (12.6)	0.001*
		18–64 years	3,538 (73.1)	1,300 (26.9)	
		Over 65	214 (58.5)	152 (41.5)	
	**Sex (n = 6,351)**	Female	2,390 (77.1)	708 (22.9)	0.001*
		Male	2,364 (72.7)	889 (27.3)	
	**Quintiles of deprivation in PCT (n = 6,259)**	Most deprived1	1,028 (69.6)	449 (30.4)	0.001*
		2	982 (74.5)	336 (25.5)	
		3	1,086 (76.8)	328 (23.2)	
		4	1,014 (77.2)	300 (22.8)	
		Least deprived 5	571 (77.6)	165 (22.4)	
	**Smoking status (n = 3,436)**	No	2,176 (77.6)	627 (22.4)	0.001*
		Yes	382 (60.3)	251 (39.7)	
**Partial denture**	**Overall**		6,027 (94.9)	324 (5.1)	
	**Adult payment status (n = 5,185)**	Exempt	915(91)	90 (9)	
		Non-exempt	3,947 (94.4)	233 (5.6)	0.001*
	**Age groups (n = 6,351)**	Under 18	1147(100)	0 (0)	0.001*
		18–64 years	4,611 (95.3)	227 (4.7)	
		Over 65	269 (73.5)	97 (26.5)	
	**Sex (n = 6,351)**	Female	2,954 (94.5)	144 (4.6)	0.109
		Male	3,073 (95.4)	180 (5.5)	
	**Quintiles of deprivation (in PCT) (n = 6,259)**	Most deprived 1	1,386 (93.8)	91 (6.2)	0.02*
		2	1,248 (94.7)	70 (5.3)	
		3	1,343 (95.0)	71 (5.0)	
		4	1,269 (96.6)	45 (3.4)	
		Least deprived 5	692 (94.0)	44 (6)	
	**Smoking status (n = 3,436)**	No	2,661 (94.9)	142 (5.1)	0.001*
		Yes	545 (86.1)	88 (13.9)	

Note

n = 6,351 unless otherwise stated

* marks statistically significant differences (p<0.05)

Treatments relate to the care delivered within closed/completed treatment plans

A higher proportion of smokers had received ‘scale and polish’ than non-smokers (68.2% cf 47.7%; p = 0.001), as had a higher proportion of non-exempt than exempt adults (47% cf 34%; p = 0.001). Analysis by deprivation status suggested that 47% of those from the least deprived quintile had received a ‘scale and polish’, compared with only 31% of those from the most deprived quintile (p = 0.001).

There was evidence of a higher rate of ‘tooth extraction’ amongst adults who were exempt payment, older (≥65 years), male and from areas of higher deprivation (p = 0.001); provision of this treatment showed a clear social gradient.

Smokers were three times *more likely* to have received ‘partial dentures’ compared with non-smokers (p = 0.001) and adults exempt payment were twice as likely to have received them compared with non-exempt. Additionally, over five times more ‘older adults’ received partial dentures compared with those of working age (26.5% cf 4.7%: p = 0.001).

### Adjusted analysis (adults only)

Further analysis, involving adjusted regression modelling on adult patient data (n = 2,782; 70%), revealed an association between adult patient characteristics and treatment, controlling for other variables. The partial denture model had the strongest predictive power (ROC = 0.83) and tooth restoration the weakest (ROC 0.64), whilst the other models were fairly good (ROC = 0.7). Each of the treatments is presented in Table 3 starting with the most common, tooth restoration.

**Table 3:** Adjusted logistic regression model predicting odds of treatment by patient characteristics between 2008/09 to 2011/12 academic years at UPDA.

Outcome (reference category in brackets^$^)	Predictor variable	Odds ratio	95% C.I. for Odds ratio		P value	ROC
			Lower	Upper		
**Tooth restoration**	Adult payment exemption	2.108	1.576	2.819	0.01*	0.6
	Age	1.02	1.015	1.025	0.01*	
(Quintile in PCT (5^$^)	Quintile in PCT(1)	1.655	1.274	2.151	0.01*	
	Quintile in PCT(2)	1.376	1.074	1.764	0.012*	
	Quintile in PCT(3)	1.145	0.904	1.449	0.262	
	Quintile in PCT(4)	1.086	0.862	1.368	0.483	
(Male^$^)	Sex	0.832	0.712	0.971	0.02*	
(Non-smoker^$^)	Smoking status	1.569	1.29	1.909	0.01*	
**Instruction and advice**	Adult payment exemption	2.198	1.506	3.207	0.001*	0.7
	Age	1.038	1.032	1.045	0.001*	
(Quintile in PCT (5^$^)	Quintile in PCT(1)	0.371	0.256	0.536	0.001*	
	Quintile in PCT(2)	0.48	0.332	0.692	0.001*	
	Quintile in PCT(3)	0.54	0.376	0.776	0.001*	
	Quintile in PCT(4)	0.608	0.421	0.879	0.009*	
(Male^$^)	Sex	1.192	0.993	1.43	0.059	
(Non-smoker^$^)	Smoking status	4.124	3.088	5.508	0.001*	
**Scale and polish**	Adult payment exemption	1.745	1.308	2.327	0.001*	0.7
	Age	1.037	1.032	1.043	0.001*	
(Quintile in PCT (5$)	Quintile in PCT(1)	0.512	0.379	0.692	0.001*	
	Quintile in PCT(2)	0.754	0.56	1.016	0.063	
	Quintile in PCT(3)	0.72	0.538	0.964	0.027*	
	Quintile in PCT(4)	0.818	0.608	1.101	0.185	
(Male^$^)	Sex	1.039	0.885	1.219	0.642	
(Non-smoker^$^)	Smoking status	1.737	1.421	2.124	0.001*	
**Tooth extraction**	Adult payment exemption	1.815	1.38	2.388	0.001*	0.7
	Age	1.033	1.028	1.039	0.001*	
(Quintile in PCT (5^$^)	Quintile in PCT(1)	1.508	1.102	2.063	0.01*	
	Quintile in PCT(2)	0.997	0.727	1.367	0.983	
	Quintile in PCT(3)	0.994	0.728	1.355	0.968	
	Quintile in PCT(4)	1.002	0.731	1.374	0.989	
(Male^$^)	Sex	0.8	0.672	0.953	0.012*	
(Non-smoker^$^)	Smoking status	2.03	1.663	2.477	0.001*	
**Partial denture**	Adult payment exemption	2.604	1.758	3.856	0.0001*	0.8
	Age	1.075	1.065	1.085	0.0001*	
(Quintile in PCT (5^$^)	Quintile in PCT(1)	1.087	0.655	1.802	0.748	
	Quintile in PCT(2)	0.813	0.484	1.365	0.433	
	Quintile in PCT(3)	0.927	0.561	1.531	0.767	
	Quintile in PCT(4)	0.704	0.412	1.205	0.201	
(Male^$^)	Sex	0.941	0.695	1.268	0.688	
(Non-smoker^$^)	Smoking status	3.142	2.277	4.337	0.0001*	

Note

n = 2,782

* marks statistically significant differences (p<0.05)

ROC = area under the curve

^$^ is reference category for categorical variables

Treatments within closed/completed treatment plans

Of the variables examined, ‘exemption from payment’ was the strongest predictor of ‘tooth restoration’, with exempt adults being twice as likely as non-exempt to receive a ‘tooth restoration’; (p = 0.001). There was a higher likelihood of receiving one or more restorations for adults in the two most deprived groups than the least deprived (65%; p = 0.01 and 37%; p = 0.012).

Increasing adult age was associated with a higher likelihood of receiving any of the common treatments. With each year of increasing age, adults were *more likely* to receive treatment as follows: partial denture (7%; p = 0.01); ‘scale and polish’ (3.7%); ‘tooth extraction’ (3%; p = 0.001) and ‘instruction and advice’ (3%; p = 0.001).

Patients identified as smokers were *more likely* to require one or more of the spectrum of treatments compared with non-smokers (p = 0.01); they were four times *more likely* to receive ‘instruction and advice’; three times *more likely* to receive a partial denture; twice as likely to receive a ‘tooth extraction’, and just under twice as likely to receive a ‘scale and polish’ (x1.7) and ‘tooth restoration’ (x1.5).

Patients exempt from patient charges were *more likely* to have received one or more of the following than those who pay charges: partial dentures (x2.6); ‘tooth restoration’ (x2); ‘instruction and advice’ (x2); ‘tooth extraction’ (x1.8); ‘scale and polish’ (x1.7). When compared with the least deprived quintile, the most deprived were *more likely* to have received the following at least once in the four-year period: tooth restoration (x1.7) and ‘tooth extraction’ (x1.5); however, they were *less likely* to have received a ‘scale and polish’ (x0.5) and ‘instruction and advice’ (x0.3). Females were 20% *less likely* to receive a tooth restoration’ (p = 0.02), and a ‘tooth extraction’ (p = 0.012) than males.

The influence of area deprivation on the multilevel model is presented in Table 4. When individual level variables were added to the model, the co-efficient remained the same as those presented in Table 3. Therefore Table 4 presents the null model of variance independently associated with the area level variable (123 LSOAs). The model suggested that 2.8% (p = 0.01) of the variance in proportion of patients who had received a ‘tooth extraction’ can be explained by LSOA. For ‘scale and polish’ this increased to 3.6% (p = 0.01) and for ‘instruction and advice’ 7% (p = 0.04).

**Table 4:** Multilevel regression models predicting treatments (Instruction and advice, ‘tooth extraction’ and ‘scale and polish’) by area of residence within closed/ completed treatment plans between 2008/09 to 2011/12 academic years at UPDA (Null models of 123 LSOAs).

Treatment	Null model	Variance Partition Coefficient (VPC)
**Instruction and advice**	Variance = 0.062 SE 0.024 (Wald 40.415, p = 0.0001). The β_0j = -0.263(0.041) p = 0.0001.	0.07*
**Tooth extraction**	Variance = 0.097, SE 0.033 (Wald 4.229, p = 0.003). The β_0j = -0.194 (0.048); p = 0.0001	0.028*
**Scale and polish**	Variance = 0.125, SE 0.034; Wald 12.505, p = 0.0001. The β_0j = -0.317 (0.048) p = 0.04	0.036*

Note

n = 2,782

* marks statistically significant differences (p<0.05)

Total number of areas 123 Lower Super Output Areas (LSOAs)

## Discussion

This is the first study to model contemporary NHS patient management data from primary dental care in England in order to predict clinical care. The findings demonstrate an association between treatment received and patients’ individual socio-demographic characteristics (demography, smoking, payment status) and context (deprivation at patients’ area of residence). These predictive relationships largely mirror population oral health needs from national epidemiological surveys [3,4], with one notable exception: ‘instruction and advice’ which relates to prevention. The study provides evidence of increasing treatment need with age, smoking, exemption from payment and deprivation status, all of which have implications for health services planning and provision.

This research has two important strengths. First, the use of patient management data provided valid accounts of the care provided, without patient recall or selection bias [21]. Second, this is the first example of what is analytically possible with NHS patient management primary dental care data and provides insight to the care delivered within a large educational primary care facility. There were, however, a number of limitations related to the structure and amount of information that could be extracted from the patient information system which need to be addressed. First, it was not possible to obtain data on presenting complaints and baseline oral health, as this information was recorded as free text, without any form of coding. Information on presenting complaints or initial oral health status would have enriched the findings by providing a full picture of the pathway towards oral health; however, data processing developments in dentistry have not yet resulted in script that can extract written text [34,35]. Even so, the data obtained were useful because of the system of practice in UPDA, which involved agreement of care plans with the patient in common with NHS contracts; thus ensuring expressed and normative needs were addressed in the treatment plans. In future, the use of assessments such as International Caries Diagnosis and Assessment System (ICDAS) [36], and Bleeding on Probing Indices (BPI), whereby the scores are recorded within the patient management system, would enable baseline oral health needs to be captured and prove useful for analysis. Second, the data were cross-sectional data which, as with all such studies, restricted analysis of temporality between treatments; for example, whether a patient was more likely to receive a ‘tooth restoration’ after ‘instruction and advice’ or vice versa, and limit researchers’ ability to establish causality [37]. It also limited the comparability to national reports, which identify treatment annually, or national surveys which provide evidence of treatment received during the lifecourse [38]. This limitation is a feature of how data are stored in the administrative system. It was, however, possible to ensure reliable accounts of receiving treatment within the cross-sectional study period as the validity was tested against a sample of clinical records. Third, and finally, the limitation of IMD to account for individual level deprivation [39]; this represents an average of the people living in an area and wealthier people may choose to live in socially deprived areas because of convenience. However, the use of area level measures can help establish whether there are factors in a person’s environment that may impact on their health. Furthermore, in this research, patient’s payment exemption status was used to provide an individual-level description of economic status and mitigate against bias. Payment exemption provides an indication of income at the time of care [9], and in this data set has shown good correlation to area level income deprivation [20]. However, as it includes pregnant and nursing women, findings relating to this variable should be examined and interpreted by sex.

The unadjusted model, which included children, confirmed differing patterns of care associated with age in line with having longer retention of teeth by adults in England [4]. This included older (≥65 years), and working age adults (18–64 years), having a higher rate of ‘tooth restoration’ (54.3% and 54.3% respectively), exceeding the volume amongst children and young people under 18 years (38%; p = 0.001). Equally this analysis validated the data, as expectedly children would not have denture treatment, and the results confirmed this with no children having received dentures. In addition, the relationships between common risks to oral health such as smoking, leading to increased dental need and thus the number of smokers that received all treatments exceeded the average for the whole study population.

All five adjusted models relating to adult dental care showed reasonably good predictive capability. The first four, namely ‘tooth restoration’, ‘instruction and advice’, ‘scale and polish’ and ‘tooth extraction’ shared the same predictors: age, smoking status, payment exemption and deprivation status. Sex was a predictor for just two of these treatment models: ‘tooth restoration’ and ‘tooth extraction’. In contrast to the four most common treatments, the adjusted model involving ‘partial dentures’ only included age and payment exemption as predictors.

Specifically, these models confirm that older patients, smokers, adults exempt from payment and from an area of higher deprivation, in particular, are more likely to receive common treatments. These findings may be explained by the patterns of need amongst adults described by successive epidemiological surveys highlighting trends in dental caries and periodontal disease by age [4,40,41], sex [4,42], and socioeconomic status [4,43,44], greater tooth loss and reliance on dentures in older people [4,45–47], and the contemporary approach to care within the service [48]. Socio-economic deprivation is well accepted as predicting self-reported dental need [4,49], and higher requests for tooth extraction have been demonstrated among deprived groups [50]. These findings suggest that in this state-funded health service, adults access care when they are at social disadvantage.

Smokers who were found to be between 1.5 and 4 times more likely to receive the treatments are at increased need because smoking is a major risk factor for periodontal disease [51,52], and there is evidence from other studies that smokers are more likely to attend the dentist more symptomatically than non-smokers regardless of deprivation status [53]; thus, they are more likely to require more treatment when they do attend.

Differences by sex in the treatments received may be explained by health seeking behaviour [54]. In this study female patients were 20% more likely to receive ‘tooth extraction’ and ‘tooth restoration’. Additionally, the impact of payment exemption on treatment as the second strongest predictor of care in most of the models, to smoking status, is worth further investigation. This relationship may partially be explained by its role as a proxy for income [20], and confirms the role of income inequality on oral health [55]. Whereas for males exemption from payments is solely income-related, for females it also covers pregnant and nursing mothers, thus questioning whether it can reliably be used as a proxy for income, an issue which should be investigated further in national data.

The association between partial dentures and age can be explained by patterns of tooth loss [56], and the fact that tooth loss increases with age, older adults not having benefitted from fluoride toothpaste in their earlier years [57], and having received more surgical than restorative care; thus, requiring dentures to replace missing teeth. The influence of payment exemption may be explained by the fact that payment removes the barrier to dentists delivering, and patients receiving, these more expensive treatment items of care.

This facility delivered a full range of routine NHS primary dental care which involved higher levels of preventive care in the form of ‘instruction and advice’ ‘fissure sealants’ and ‘fluoride varnish’ [58], than primary care nationally. Overall, almost half of the patients at UPDA received prevention in the form of ‘instruction and advice’ and this high rate can be explained by organisational philosophy, since UPDA is an educational institution which delivers contemporary care and embraces dental team skill mix [58]. Furthermore, this approach is supported by an NHS contract [59], which includes key performance indicators including the identification and direction of smokers to tobacco cessation services [60]. The latter explains the higher receipt of ‘instruction and advice’ for smokers. In the past, prevention has been a reflection of financial incentives [61], and poorly incentivised prevention has resulted in it being relegated to lower priority [62–64]. Thus, there is evidence that ‘instruction and advice’ was prioritised amongst the adult smokers, suggesting that performance indicators may provide an incentive for change. The most controversial finding from the study, however, was that adults from areas of higher deprivation were *less likely* to receive prevention in the form of ‘instruction and advice’ as their less deprived counterparts in the adjusted model; instead there was a clear inverse social gradient in relation to need. This is surprising as the institutional philosophy supported preventive care and high levels of prevention were delivered overall; however, additional factors such as individual practitioner and patient attitudes to the delivery and receipt of care should be considered [62].

There is evidence that even when practitioners might wish to deliver prevention to those who need it, most of these patients present late in the disease process and require emergency care [62], with the majority failing to return for prevention. Equally there is evidence of high needs patients having poor prioritisation of health care seeking [53], and of patients attending for emergency care being *less likely* to receive prevention [65]. It has to be remembered that one of the limitations of this data set was the inability to differentiate between completed and closed courses of care. Perhaps patients attending UPDA attended on an emergency basis when it may be less acceptable to receive prevention and others who did not complete their prescribed course of care missed out on preventive advice; thus, suggesting that the health behaviours of patients may widen inequalities? Further research, subject to better coding being possible within the patient management system, is required to examine the differences in care between those who complete care and those who do not. This should ideally be supplemented by exploring the views of such patients on what they want and don’t want from dental care and why.

Through the multilevel modelling this study suggests that within small residential areas (LSOAs) there are further influences which explain 7% of the variance in proportions of ‘partial dentures’, and 3% and 4% for ‘tooth extraction’ and ‘scale and polish’ respectively. This may relate to the influence of peers and social norms, but is worth further investigation. These findings parallel a study by Jamieson et al, who showed that variance in dental caries experience was associated with residence [35]. Further research examining the influence of local environments is necessary to uncover protective and risk factors to oral health in the environment.

This research has a number of implications. First, it has highlighted the importance of maximising the use and enhancing the quality of routinely collected data and the need to improve storage mechanisms to enable longitudinal analysis to further develop our understanding of care patterns and health. This should include specific enhancement to ensure the inclusion of relevant indices and coded data to provide patient baseline data, and in-time outcomes. Second, these results provide insight into the provision and receipt of contemporary dental care, and should inform discussions regarding performance indicators that target priority groups such as smokers and future planning for our ageing population. Third, it could be argued that despite a system of subsidised care, people from areas of high deprivation had more extractions, and experienced inequality in prevention and restoration which is likely to increase the gap in oral health between deprived and affluent sections of society. The implication is that this inverse social gradient in relation to ‘instruction and advice’ must be investigated further to explore the balance of patient, practitioner, organisational and system issues to ensure that inequalities do not widen. There is also a need to identify factors that hamper or encourage preventive care for adults, in order to fulfil the NHS priority to make every encounter count for prevention [66]. Fourth, the role of payment exemption requires further investigation to understand whether this variable acts as a proxy for income deprivation over time, particularly between the sexes. Fifth, and finally, further research is needed on environmental factors that may contribute to dental care.

## Conclusion

This is the first study to model patient management data from a state-funded dental service and show that individual and contextual factors predict common treatments received. Implications of this research include the importance of making provision for our aging population and ensuring that preventative care is available to all. Further research is required to explain the interaction of organisational and system policies, practitioner and patient perspectives on care and, thus, inform effective commissioning and provision of dental services.

## Supporting Information

S1 Dataset (SAV)
